# Short stature-related single-nucleotide polymorphism (SNP) activates endothelial repair activity in elderly Japanese

**DOI:** 10.1186/s12199-019-0780-1

**Published:** 2019-05-01

**Authors:** Yuji Shimizu, Hirotomo Yamanashi, Yuko Noguchi, Jun Koyamatsu, Mako Nagayoshi, Kairi Kiyoura, Shoichi Fukui, Mami Tamai, Shin-Ya Kawashiri, Kazuhiko Arima, Takahiro Maeda

**Affiliations:** 1 Department of Community Medicine, Nagasaki University Graduate School of Biomedical Sciences, Nagasaki-shi, Sakamoto 1-12-4, Nagasaki, 852-8523 Japan; 2 Department of Cardiovascular Disease Prevention, Osaka Center for Cancer and Cardiovascular Disease Prevention, Osaka, Japan; 3 Department of General Medicine, Nagasaki University Hospital, Nagasaki, Japan; 4 Department of Island and Community Medicine, Nagasaki University Graduate School of Biomedical Sciences, Nagasaki, Japan; 5 Department of Immunology and Rheumatology, Nagasaki University Graduate School of Biomedical Sciences, Nagasaki, Japan; 6 Department of Public Health, Nagasaki University Graduate School of Biomedical Sciences, Nagasaki, Japan

**Keywords:** Height, Hypertension, Platelets, rs3782886, SNP

## Abstract

**Background:**

Hypertension and atherosclerosis are bidirectionally related, while platelet count could serve as an indicator of endothelial repair. Therefore, high platelet counts could be associated with hypertension by indicating more intense endothelial repair activity. Furthermore, short stature has been shown to constitute a risk of atherosclerosis. Since inflammation-related single-nucleotide polymorphism (SNP (rs3782886)) is reportedly associated with myocardial infarction and short stature, rs3782886 could be associated with a high platelet count and thus more intense endothelial repair activity.

**Methods:**

We conducted a cross-sectional study of 988 elderly Japanese who participated in a general health check-up. Short stature was defined as a height of at or under the 25th percentile of the study population, and high platelet count as the highest tertiles of the platelet levels.

**Results:**

High platelet counts were found to be independently and positively associated with hypertension while rs3782886 was independently associated with high platelet levels and short stature. The classical cardiovascular risk factor-adjusted odds ratio (OR) and 95% confidence interval (CI) of high platelet count for hypertension was 1.34 (1.02, 1.77). With non-minor homo of the rs3782886 as the reference group, the adjusted OR and 95% CI for high platelet count and short stature of minor home were 2.40 (1.30, 4.42) and 2.21 (1.16, 4.21), respectively.

**Conclusion:**

SNP (rs3782886) was shown to be associated with high platelet count and short stature. This result partly explains how a genetic factor can influence the impact of height on endothelial repair.

## Background

Hypertension is known as a major risk factor of stroke for Japanese [1] while short stature is well known as a Japanese characteristic. Furthermore, short stature has been reported to have an independent association with hypertension [2], carotid atherosclerosis [3], and incidence of stroke [4]. However, previous studies of ours indicate that the presence of carotid atherosclerosis cannot explain the risk of stroke associated with short stature [3, 4].

Recently, hematopoietic activity has been revealed to be closely associated with vascular maintenance activity (endothelial repair activity [5–8]). Short stature has been shown to be associated with lower capacity for hematopoietic activity [9, 10] and with anemia [11] possibly due to the fact that height indicates the absolute volume of total bone marrow. Higher capacity for hematopoietic activity (high hemoglobin level) has been found to be positively associated with atherosclerosis [6, 7] and hypertension [8, 12].

Those studies raise issues emerging from paradoxical findings for short stature as a risk for anemia, hypertension, carotid atherosclerosis, and incidence of stroke.

Since atherosclerosis results from aggressive endothelial repair, the findings of these studies indicate that the risk of stroke in people with short stature might be associated with low endothelial repair activity (low hematopoietic activity).

Single-nucleotide polymorphism (SNP) (rs3782886) in breast cancer suppressor BRCA1-related associated protein (BRAP) is reportedly associated with risk of myocardial infarction [13], while SNP (rs3782886) is also found to be associated with short stature in a previous study by us [14]. Another study reported that BRAP activates inflammatory cascades and increases the risk of carotid atherosclerosis [15] but is not associated with risk of stroke [16]. Although an SNP (rs3782886) could be associated with carotid atherosclerosis, these studies indicate that it might also have a beneficial effect on stroke prevention by inducing endothelial repair in participants with short stature; otherwise, these participants would have high risks of hypertension [2], carotid atherosclerosis [3], and stroke [4].

Furthermore, platelet is known to contribute to endothelial repair [17]. We previously showed that platelet levels were positively associated with hypertension [18] and that platelet count could serve as an indicator of vascular repair (endothelial repair and developing atherosclerosis) [19]. Since the association between hypertension and atherosclerosis (increased arterial stiffness) is bidirectional [20–22], high platelet levels, which are associated with hypertension, can be expected to reflect a higher activity of endothelial repair.

Since height is inversely associated with platelet count in elderly men [23], clarification of the relationship of SNP (rs3782886) with short stature and high platelet counts, which are in turn associated with hypertension, should provide us with an efficient tool to clarify the underlying mechanism of the effect of height on endothelial repair.

We therefore hypothesized that hypertension is positively associated with a high platelet count, thus indicating active endothelial repair. Moreover, that SNP (rs3782886) is also associated with a high platelet count and short stature since it plays an important role in the activation of endothelial repair in participants with short stature.

To elucidate those associations, we conducted a cross-sectional study of 988 elderly Japanese participants aged 60–89 years who participated in a general health check-up in 2014 and 2015.

## Methods

### Study population

The total number of residents of Goto City aged 60–89 in 2015 was 16,176, based on an estimate by the National Institute of Population and Social Security Research in March 2013 [24].

The study population comprised 992 Japanese elderly residents aged 60–89 years from the rural communities of the Goto Islands in western Japan, who underwent annual medical check-ups in 2014 and 2015 as recommended by the Japanese government.

Participants without SNP data (*n* = 3) and without blood pressure data (*n* = 1) were excluded, leaving 988 participants with a mean age of 72.6 years (standard deviation (SD), 7.3; range, 60–89) enrolled in the study.

### Data collection and laboratory measurements

Trained interviewers obtained information on medical history (lifestyle habit). Body weight and height were measured with an automatic body composition analyzer (BF-220; Tanita, Tokyo, Japan), and body mass index (BMI; kg/m^2^) was calculated. Systolic and diastolic blood pressure were recorded at rest. Blood samples were collected in an EDTA-2 K tube, a siliconized tube, and sodium fluoride tube. The number of platelets in samples from the EDTA-2 K tube was measured with an automated procedure at SRL, Inc. (Tokyo, Japan). Triglycerides (TG) and serum creatinine were measured enzymatically, while HDL cholesterol (HDLc) was measured with a direct method and hemoglobin A1c (HbA1c) with the latex coagulation method. Genomic DNA was extracted from 2 ml of whole peripheral blood by means of the Gene Prep Star NA-480 (Kurabo Industries Ltd., Osaka, Japan) and typed for SNP rs3782886 (BRAP on chromosome 12q24.12) by using the HybProbe method with LightCycler 480 (Roche Diagnostics, Basel, Switzerland). Hypertension was defined as systolic blood pressure ≥ 140mmHg and/or diastolic blood pressure ≥ 90 mmHg. A high platelet level was defined as the highest tertile (≥ 22.0 × 10^4^/μL for men and ≥ 24.2 × 10^4^/μL for women) and short stature as a height level at or under the 25th percentile of the study population (< 158.4 cm for men and < 146.4 cm for women) as in a previous study by us [3, 14].

### Statistical analysis

Characteristics of the study population stratified by platelet level and SNP (rs3782886) genotype were expressed as mean ± standard deviation. A trend test was performed with analysis of variance (ANOVA) for continuous values, and chi-squared test was used for determining proportions.

Logistic regression models were also used to calculate odds ratios (ORs) and 95% confidence intervals (CIs) in order to determine the influence of platelet level on hypertension. In addition, ORs and 95% CIs were calculated by using a logistic regression model to determine the influence of SNP (rs3782886) on high platelet counts, hypertension, and short stature.

Adjustments for confounding factors were made by using two models. In the first model (model 1), adjustment was made only for age and sex. Alcohol consumption has been shown to be positively associated with height in previous studies [3, 4, 25], and white blood cell count to be a factor that is influenced by smoking status [26] while height is inversely associated with high white blood cell count [25]. BMI could be positively associated with endothelial repair (CD34-positive cell count) [8, 18, 27–29] while height is positively associated with CD34-positive cell count in participants with systolic hypertension [30]. Another study reported the identification of a primary association between a genetically determined short stature and an increased risk of cardiovascular disease which may be partly explained by the association between short stature and adverse lipid profile [31]. In one of our studies, we reported that the activity of endothelial repair (CD34-positive cells) might influence the association between blood pressure and TG [29] and HDLc [28]. HbA1c has also been found to be associated with atherosclerosis [32, 33]. All these factors are well-known classical cardiovascular risk factors. Furthermore, renal function is reportedly associated with SNP (rs3782886) [34], endothelial repair (CD34-positive cells) [35], anemia [36], and incidence of stroke [37]. We therefore added serum creatinine as a confounding factor to the aforementioned classical cardiovascular risk factors for the present analysis. Therefore, the second model (model 2) included other, possibly confounding, factors, namely, BMI (kg/m^2^), (systolic blood pressure (mmHg), which was not applicable to the analysis of the associations between platelet count and hypertension nor to the associations between SNP (rs3782886) and hypertension), alcohol consumption (never-drinker, former drinker, current drinker [< 23 g/week, 23–46 g/week, 47–68 g/week, ≥ 69 g/week]), smoking status (never-smoker, former smoker, current smoker), HDLc (mg/dL), TG (mg/dL), HbA1C (%), and serum creatinine (mg/dL).

For the sensitivity analysis, we repeated our investigations with sex-specific models.

All statistical analyses were performed with the SAS system for Windows (version 9.4; SAS Inc., Cary, NC). Values of < 0.05 were regarded as being statistically significant.

## Results

### Characteristics of study population in relation to platelet levels

Characteristics of the study population in relation to platelet levels are shown in Table 1. The current smoker group showed positive associations with platelet levels while age and serum creatinine were inversely associated with platelet levels.

**Table 1:** Characteristics of the study population in relation to platelet levels

	Platelet levels			*P*
	T1 (low)	T2	T3 (high)	
No. of participants	332	327	329	
Age, years	73.6 ± 7.1	72.4 ± 7.3	71.6 ± 7.4	0.003
Gender of men, %	37.0	37.6	37.4	0.989
Body mass index (BMI), kg/m^2^	23.1 ± 3.6	23.3 ± 3.1	23.2 ± 3.2	0.776
Systolic blood pressure, mmHg	139 ± 18	139 ± 17	141 ± 19	0.179
Diastolic blood pressure, mmHg	80 ± 11	81 ± 11	82 ± 11	0.080
Current drinker, %	22.6	23.5	27.4	0.323
Current smoker, %	3.3	6.4	8.8	0.013
Serum triglycerides (TG), mg/dL	93 ± 54	108 ± 63	106 ± 53	0.001
Serum HDL cholesterol (HDLc), mg/dL	59 ± 16	60 ± 18	60 ± 15	0.565
Hemoglobin A1c (HbA1c), %	5.7 ± 0.5	5.6 ± 0.4	5.8 ± 0.6	0.002
Serum creatinine, mg/dL	0.78 ± 0.22	0.75 ± 0.19	0.73 ± 0.18	0.003
Platelets, × 10^4^/μL	16.6 ± 2.7	21.5 ± 1.4	27.7 ± 5.1	< 0.001

Values: mean ± standard deviation. Platelet levels for men are < 18.7 × 10^4^/μL for T1, 18.7–21.9 × 10^4^/μL for T2, and ≥ 22.0 × 10^4^/μL for T3, for women are < 20.3 × 10^4^/μL for T1, 20.3–24.1 × 10^4^/μL for T2, and ≥ 24.2 × 10^4^/μL for T3

### Association between platelet levels and hypertension

Table 2 shows ORs and 95% CIs for hypertension in relation to platelet count. Independent of known cardiovascular risk factors, the high platelet level group showed significantly higher OR for hypertension compared with the reference group of non-high platelet levels (T1 and T2); the adjusted OR and 95% CI for hypertension was 1.34 (1.02, 1.77).

**Table 2:** Odds ratios (ORs) and 95% confidence intervals (CIs) for hypertension in relation to platelet levels

	Platelet levels			*P* for trend	1 SD increment of platelet
	T1 (low)	T2	T3 (high)		
No. at risk	332	327	329		
No. of cases (percentage)	160 (48.2)	170 (52.0)	185 (56.2)		
Model 1	1.00	1.22 (0.89, 1.66)	1.49 (1.09, 2.04)	0.012	1.16 (1.02, 1.33)
	1.00		1.35 (1.03, 1.77)	0.029	
Model 2	1.00	1.20 (0.87, 1.64)	1.47 (1.07, 2.03)	0.018	1.15 (1.01, 1.32)
	1.00		1.34 (1.02, 1.77)	0.036	

*Model 1* adjusted only for sex and age. *Model 2* further adjusted for body mass index, alcohol consumption (never-drinker, former drinker, current drinker [< 23 g/week, 23–45 g/week, 46–68 g/week, ≥ 69 g/week]), smoking status (never-smoker, former smoker, current smoker), HDL cholesterol, triglycerides, HbA1C, and serum creatinine. Hypertension is defined as systolic blood pressure ≥ 140 mmHg and/or diastolic blood pressure ≥ 90 mmHg. Platelet levels for men are < 18.7 × 10^4^/μL for T1, 18.7–21.9 × 10^4^/μL for T2, and ≥ 22.0 × 10^4^/μL for T3, for women are < 20.3 × 10^4^/μL for T1, 20.3–24.1 × 10^4^/μL for T2, and ≥ 24.2 × 10^4^/μL for T3. 1 standard deviation (SD) increments of platelet are 6.23 × 10^4^/μL for men and 5.26 × 10^4^/μL for women

### Characteristics of the study population by genotype of SNPs (rs3782886)

Characteristics of the study population by genotype of SNPs (rs3782886) are shown in Table 3. Of the 988 elderly Japanese participants, 609 were major homogeneous (A/A), 332 were heterogeneous (A/G), and 47 were minor homogeneous (G/G). This SNP was in Hardy-Weinberg equilibrium. Current drinker was inversely significantly associated with minor allele frequency (G) while platelet level showed a significantly positive association.

**Table 3:** Characteristics of the study population by genotype of rs3782886

	rs3782886			*P*
	Major homo (A/A)	Hetero type (A/G)	Minor homo (G/G)	
No. of participants	609	332	47	
Age, years	72.1 ± 7.2	73.2 ± 7.4	72.8 ± 7.1	0.089
Gender of men, %	36.3	40.1	31.9	0.382
Body mass index (BMI), kg/m^2^	23.1 ± 3.2	23.4 ± 3.5	23.0 ± 3.3	0.274
Systolic blood pressure, mmHg	140 ± 18	140 ± 18	139 ± 19	0.990
Diastolic blood pressure, mmHg	82 ± 11	80 ± 11	81 ± 11	0.134
Current drinker, %	32.7	13.0	0.0	< 0.001
Current smoker, %	7.2	4.8	2.1	0.171
Serum triglycerides (TG), mg/dL	102 ± 60	103 ± 53	100 ± 39	0.941
Serum HDL cholesterol (HDLc), mg/dL	61 ± 17	58 ± 15	60 ± 18	0.066
Hemoglobin A1c (HbA1c), %	5.7 ± 0.5	5.7 ± 0.5	5.7 ± 0.6	0.722
Serum creatinine, mg/dL	0.75 ± 0.20	0.77 ± 0.19	0.75 ± 0.20	0.149
Platelets, × 10^4^/μL	21.5 ± 4.9	22.4 ± 6.9	23.2 ± 4.9	0.019

Values: mean ± standard deviation

### Associations between rs3782886 genotype and high platelet levels

Table 4 shows the associations between genotype and high platelet levels. For model 1 with major homogeneity (A/A) as the reference group, no significant association was observed for heterogeneity (A/G), while a significant association was observed for minor homogeneity (G/G). When we made further adjustment for other known cardiovascular risk factors, those associations with the reference group became slightly stronger and the statistical value became significant for heterogeneity.

**Table 4:** Odds ratios (ORs) and 95% confidence intervals (CIs) for high platelet in relation to rs3782886 genotype

	rs3782886			*P* for trend
	Non-minor homo		Minor homo (G/G)	
	Major homo (A/A)	Hetero (A/G)		
No. at risk	609	332	47	
No. of cases (percentage)	186 (30.5)	119 (35.8)	24 (51.1)	
Model 1	1.00	1.31 (0.99, 1.74)	2.44 (1.34, 4.45)	0.002
	1.00		2.21 (1.22, 3.99)	0.009
Model 2	1.00	1.49 (1.10, 2.01)	2.86 (1.52, 5.37)	< 0.001
	1.00		2.40 (1.30, 4.42)	0.005

*Model 1* adjusted only for sex and age. *Model 2* further adjusted for body mass index, systolic blood pressure, alcohol consumption (never-drinker, former drinker, current drinker [< 23 g/week, 23–45 g/week, 46–68 g/week, ≥ 69 g/week]), smoking status (never-smoker, former smoker, current smoker), HDL cholesterol, triglycerides, HbA1C, and serum creatinine. The high platelet level is defined as the highest tertiles of platelets levels (≥ 22.0 × 10^4^/μL for men and ≥ 24.2 × 10^4^/μL for women)

### Associations between the rs3782886 genotype and short stature

Table 5 shows the associations between genotype and short stature. With major homogeneity (A/A) as the reference group, no significant association was observed for heterogeneity (A/G), while a significant association was observed for minor homogeneity (G/G). However, a comparison of the two categories non-minor homogeneity and minor homogeneity showed a significant association.

**Table 5:** Odds ratios (ORs) and 95% confidence intervals (CIs) for short stature in relation to rs3782886 genotype

	rs3782886			*P* for trend
	Non-minor homo		Minor homo (G/G)	
	Major homo (A/A)	Hetero (A/G)		
No. at risk	609	332	47	
No. of cases (percentage)	132 (21.7)	94 (28.3)	19 (40.4)	
Model 1	1.00	1.32 (0.96, 1.83)	2.55 (1.33, 4.89)	0.004
	1.00		2.30 (1.21, 4.36)	0.011
Model 2	1.00	1.33 (0.94, 1.87)	2.51 (1.29, 4.89)	0.006
	1.00		2.21 (1.16, 4.21)	0.016

*Model 1* adjusted only for sex and age. *Model 2* further adjusted for body mass index, systolic blood pressure, alcohol consumption (never-drinker, former drinker, current drinker [< 23 g/week, 23–45 g/week, 46–68 g/week, ≥ 69 g/week]), smoking status (never-smoker, former smoker, current smoker), HDL cholesterol, triglycerides, HbA1C, and serum creatinine. Short stature is defined as a height level at or under the 25th percentile of the study population (< 158.4 cm for men and < 146.4 cm for women)

### Associations between rs3782886 genotype and hypertension

No significant associations between genotype and hypertension were observed (Table 6).

**Table 6:** Odds ratios (ORs) and 95% confidence intervals (CIs) for hypertension in relation to rs3782886 genotype

	rs3782886			*P* for trend
	Non-minor homo		Minor homo (G/G)	
	Major homo (A/A)	Hetero (A/G)		
No. at risk	609	332	47	
No. of cases (percentage)	320 (52.5)	170 (51.2)	25 (53.2)	
Model 1	1.00	0.91 (0.70, 1.20)	1.00 (0.55, 1.82)	0.652
	1.00		1.03 (0.57, 1.87)	0.912
Model 2	1.00	0.93 (0.70, 1.23)	1.05 (0.57, 1.93)	0.324
	1.00		1.08 (0.60, 1.98)	0.791

*Model 1* adjusted only for sex and age. *Model 2* further adjusted for body mass index, alcohol consumption (never-drinker, former drinker, current drinker [< 23 g/week, 23–45 g/week, 46–68 g/week, ≥ 69 g/week]), smoking status (never-smoker, former smoker, current smoker), HDL cholesterol, triglycerides, HbA1C, and serum creatinine. Hypertension is defined as systolic blood pressure ≥ 140 mmHg and/or diastolic blood pressure ≥ 90 mmHg

The sensitivity analysis showed similar associations to those obtained for the main results.

## Discussion

The major findings of the present study with elderly Japanese participants are that a high platelet level is positively associated with hypertension for elderly Japanese participants and that inflammation-related SNP (rs3782886) is significantly associated with a high platelet level and short stature.

Recently, platelets have been revealed to play a major role in inflammation, as well as to be important as an initial activator for the development of atherosclerotic lesions [38]. Even though development of atherosclerosis is only one aspect of endothelial repair, the association between hypertension and endothelial dysfunction (atherosclerosis) is bidirectional: hypertension induces increased arterial stiffness and vice versa [20–22]. Since our study presented here discovered a significant positive association between high platelet levels and hypertension, the former can be assumed to indicate a greater level of endothelial repair activity. In one of our previous studies, a high platelet count was found to be positively associated with hypertension in elderly men [18]. We also found that platelet levels are positively associated with carotid intima-media thickness (CIMT) in elderly hypertensive men [18, 19]. Those findings also support our hypothesis that a high platelet count is likely to indicate a higher level of endothelial repair activity. In addition, SNP (rs3782886) is known to be located in the BRAP gene on chromosome 12q24 and that a higher expression of the BRAP minor allele is associated with an increased risk of atherosclerosis by heightening the degree of inflammation through activation of the NF-κB protein [15, 39]. Since activation of the NF-κB pathway also could promote platelet activation protein [40], our finding that SNP (rs3782886) is significantly associated with high platelet levels is likely to indicate that the genetic factor might influence the level of endothelial repair activity.

Furthermore, short stature has been shown to constitute an inflammatory risk for elderly Japanese men as shown by a high white blood cell count [25]. Moreover, SNPs (rs3782886) were shown to be associated with short stature in a previous study by us [14]. SNPs (rs3782886) were also found to be significantly associated with a high platelet count and short stature in the current study, indicating that the genetic factor may at least partly influence the risk of inflammatory changes which induce endothelial repair in participants with short stature. Another study by us of elderly men also supports the notion of this mechanism. In this study, we showed that platelet level is positively associated with CIMT and circulating endothelial progenitor cells (CD34-positive cells) while height is inversely associated with platelet count for participants with high hemoglobin levels [23].

The inverse association between height and hypertension which is reported in a Chinese study [2] is probably due to a reduced anti-oxidative stress capacity [20, 21, 41] since short stature is associated with lower hematopoiesis activity [9, 10] and higher risk of anemia [11]. Lower endothelial repair capacity which is a characteristic of short stature [23, 30] also might induce hypertension because of its reduced ability to compensate for blockage of the blood flow. Moreover, hypertension is a well-known major risk factor for stroke in Japanese [1] who are known to be characterized by short stature and low BMI. Since we reported in a previous study that height was inversely associated with the incidence of stroke only in participants with low BMI (< 23 kg/m^2^) [4], and that carotid atherosclerosis was inversely associated with height only in participants with high BMI (≥ 25 kg/m^2^) [3], carotid atherosclerosis alone could not explain the risk of stroke for Japanese with short stature. In fact, reduced capability for endothelial repair [23, 30] and hypertension [2] characteristic of short stature might perform an important role in the incidence of stroke for participants with short stature. Since BRAP is reportedly associated with atherosclerosis [15] but not associated with stroke [16] and SNPs (rs3782886) are associated with short stature [14], SNP (rs3782886) might perform an import function for the prevention of stroke for participants with short stature by stimulating endothelial repair. In the present study, even though SNP (rs3782886) was found to be associated with a high platelet count, which is related to hypertension, no significant association between SNP (rs3782886) and hypertension was observed, which partly supports the aforementioned mechanisms because adequate endothelial maintenance was performed predominantly for normal body weight participants. To clarify those mechanisms, further study is needed with a larger population which makes it possible to perform a BMI status-specific analysis of the relationship between SNP (rs3782886) and hypertension.

From the anthropological point of view, an extensive presence of SNP (rs3782886) should have some beneficial effects on life activities rather than disadvantages. Even though SNP (rs3782886) is reportedly associated with myocardial infarction [13], the findings presented here indicate that the beneficial effect of SNP (rs3782886) is a high endothelial repair activity among participants with short stature whose endothelial repair activity is low [9, 10, 23].

The present findings are efficient for clarifying the mechanism underlying the association between height and endothelial repair as they indicate that genetic factors could at least partly influence this association. In addition, the present findings also clarify the reason why the extensive presence of inflammatory disadvantage-associated SNP (rs3782886) [13, 15] could be established among the general Japanese population.

Possible limitations of this study warrant consideration. First, because creatinine clearance data were not available and estimated glomerular filtration rate (GFR) is not an effective tool for evaluating kidney function for a comparison of associations with various body heights [3, 4, 9, 10, 25, 42], we could not perform an analysis adjusted for accurate renal function. However, our study showed that associations, such as between high platelet and hypertension, SNPs (rs3782886) and high platelet level, and SNPs (rs3782886) and short stature, remained significant even after adjustment for serum creatinine. Second, because this was a cross-sectional study, causal relationships were not able to be established. Furthermore, because our study used elderly Japanese who participated in a general health check-up, selection bias arising from this kind of study is unavoidable.

## Conclusion

In conclusion, there is a significant and positive association between high platelet count and hypertension, and SNPs (rs3782886) were shown to be associated with high platelet count and short stature. These results partly explain how a genetic factor can influence the impact of height on endothelial repair.

## Abbreviations

ANOVA: Analysis of variance
BMI: Body mass index
BRAP: BRCA1-related associated protein
CI: Confidence interval
CIMT: Carotid intima-media thickness
GFR: Glomerular filtration rate
HbA1c: Hemoglobin A1c
HDLc: HDL cholesterol
OR: Odds ratio
SD: Standard deviation
SNP: Single-nucleotide polymorphism
TG: Triglycerides
